# Effects of Genotype and Culture Conditions on Microspore Embryogenesis and Plant Regeneration in *Brassica Rapa* ssp. *Rapa* L.

**DOI:** 10.3390/plants9020278

**Published:** 2020-02-21

**Authors:** Daria Shumilina, Dmitry Kornyukhin, Elena Domblides, Alexey Soldatenko, Anna Artemyeva

**Affiliations:** 1 Federal State Budgetary Scientific Institution Federal Scientific Vegetable Center, Selektsionnaya St., 14, VNIISSOK, Odintsovo Reg., Moscow District 143072, Russia; 2 Federal Research Center the N.I. Vavilov All-Russian Institute of Plant Genetic Resources, 42,44, B. Morskaya Street, St. Petersburg 190000, Russia

**Keywords:** *Brassica rapa*, microspore culture, doubled haploid, microsporogenesis disorder

## Abstract

Turnip is a biennial crop and, consequently, the creation of pure lines for breeding is a time-consuming process. The production of pure turnip lines using doubled haploids produced in isolated microspore culture has not been sufficiently developed. The aim of the present work was to determine some key factors inducing embryogenesis in the isolated microspore culture of turnip, as well as investigating the manners of embryo development. It was shown that the acidity of the medium is an important factor in embryo production; different optimal pH levels ranging from 6.2 to 6.6 corresponded to individual genotypes. Such factors as the cold treatment of buds and the addition of activated charcoal to the nutrient medium increased the responsiveness of all genotypes studied. The turnip variety ‘Ronde witte roodkop herfst’ demonstrated a genetic disorder in the development of microspores; namely, non-separation of some microspores from tetrads. In the in vitro culture, each of the daughter microspores developed on its own. This indicates the dependence of the possibility of embryogenesis in the turnip microspore culture on the genotype. Results suggest that the initiation of secondary embryogenesis in primary embryos leads to an increase in the proportion of doubled haploid plants.

## 1. Introduction

Turnip is a crop which has been traditionally cultivated in Russia since ancient times. Before the beginning of potato cultivation in Russia (in the 18th century), it was one of the staple food crops. Traditionally, turnip varieties have been created by crossing and long-term selection aimed at obtaining a genetically homogenous population. The modern technology of doubled haploid line production through microspore culture allows us to quickly obtain homozygous lines. Such lines are very important for breeding; furthermore, they serve as useful tools for genetic and molecular research in plants. Many articles have been published on various species of the Brassicaceae family since Lichter’s report [1] about the successful isolation of a *Brassica napus* microspore culture. However, a small number of embryos in the culture of turnip microspores [2], as well as a large dependence on the genotype, have been noted. These circumstances have been shown to prevent the production of doubled haploid lines for a large number of varieties [3]. Dependence on heat stress, sucrose concentration, and acidity of the medium, which influence the ability to switch to the sporophytic path of development in the microspore culture, is common in cabbage crops. The other group of factors determines the direction of cell differentiation (i.e., direct embryogenesis through the formation of suspensor-like structures or a callus). These factors are the microspore development stage, heat shock duration and temperature, acidity of the medium, and plant growth regulators. Studies have shown that cold treatment of flower buds, as well as optimal acidity [4], enhance the induction of embryogenesis [5]. Zhang et al. [6] found that temperature shock and the application of plant growth regulators can optimize the isolated microspore culture protocol for turnips.

Contradictions have been found in recommendations concerning the use of activated charcoal (AC). Researchers have noted the negative impact of AC on embryo formation in the absence of agarose and in the presence of growth and development regulators [7,8]. In other studies, the application of AC together with agarose significantly enhanced the frequency of embryogenesis and the rate of successful embryo development [9,10,11]. The data on embryo growth are also controversial. Most studies have been aimed at stimulating direct embryo-to-plantlet conversion. This has been achieved through the application of abscisic acid and embryo drying [12,13,14,15]. Studies on *Brassica rapa* ssp. *chinensis* [16] showed that the percentage of haploid plantlet formation after direct regeneration was higher than in the case of development from secondary embryos, thus increasing the effectiveness of the biotechnological approach and making it possible to do without colchicine application.

The aim of the present study was to investigate the microspore embryogenesis and in vitro double haploid plant development in cultivars of turnip (*Brassica rapa* ssp. *rapa* L.). Special attention was paid to the development of a protocol for choosing microspore cultivation conditions to obtain the highest number of embryos for each genotype, considering the effects of AC, acidity level, and plant growth, as well as development regulators in the culture medium.

## 2. Results

### 2.1. The Dependence of Embryogenesis in the Turnip Microspore Culture on the Genotype

It is well attested in the literature that the use of the oilseed rape haploid plant production protocol for turnips leads to a low yield of doubled haploid (DH)-regenerated plants. Therefore, it has been a priority to determine the basic parameters of the cultivation technique for turnips. Four turnip varieties, differing by origin and taken from the collection of the N.I. Vavilov All-Russian Institute of Plant Genetic Resources, were used in this study (see Table 1). In the first set of experiments, standard microspore culture parameters (½ NLN13 medium, pH 5.8) were used, without flower bud pre-treatment or AC application.

Differences in the responsiveness of individual genotypes in turnip microspore culture were clearly observed. Formation of embryos, calluses, and (further on) of normally developing regenerated plants in the microspore culture of different genotypes fluctuated by more than 10 times (Table 1). Plants of the Roots turnip variety were the most responsive. More than 20 embryos of this variety were obtained per one Petri dish. In the case of the Brassicaceae family, formation of only embryos or suspensor-like structures usually occurs in the microspore culture in the absence of plant growth regulators. The formation of callus in turnip microspore culture was also observed. The intensity of callus formation depended on the plant genotype.

### 2.2. Optimisation of Microspore Culture Parameters for Embryo Production

Changes in the culture medium composition and selection of suitable cultivation conditions lead to the formation of embryos even in the unresponsive genotypes of cabbage crops [16,17]. The responsiveness of turnip microspores to changes in the medium composition was tested using the most responsive turnip variety (Roots). During the experiments, modifications of the original protocol were made. Activated charcoal (AC) was added to the liquid medium to reduce the negative impact of undesirable substances, such as metabolites of growing embryos or microspore debris. The effectiveness of several variants of the medium pH and of plant growth regulators in the successful development of plantlets regenerating from embryos and in the increase in turnip DH yield were tested. Finally, the effect of cold pre-treatment at 10 °C on turnip flower buds used for microspore isolation was investigated.

**Figure 1:**
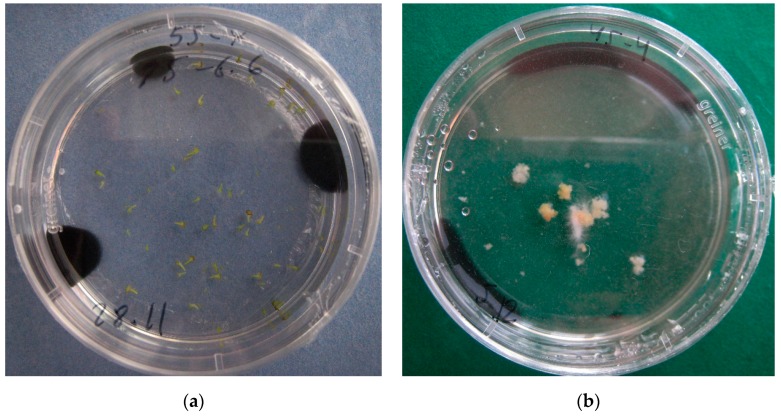
Types of turnip microspores development in the in vitro culture: (**a**) embryos of the ‘Roots’ variety, medium pH 6.6 and AC presence; (**b**) calluses and embryos of variety ‘York Globe’, medium pH 6.6 and AC presence.

It was found that the application of AC increased the yield of embryos at all medium pH values. Significantly fewer embryos formed in the medium without AC. At a pH value of 6.6, the embryo yield was the highest for the Roots variety, amounting to over 50 pieces per Petri dish (Table 2 and Figure 1a).

Tests of medium variants differing in pH level and AC presence/absence made it possible to choose the optimal condition for each tested turnip variety (Tables S1–S3 Supplementary Materials). Unfortunately, undesirable callus formation was found in the microspore cultures of the Snowball and York Globe varieties (Table 3 and Figure 1b).

Cold pre-treatment of flower buds has been used to increase the yield of embryos in oilseed rape and other cabbage crops. In a similar way, turnip flower buds were pre-treated at 10 °C for 48 h. This treatment increased the number of dividing microspores for all varieties. In the case of Snowball and York Globe varieties, temperature shock led to an increase in embryo formation and a decrease in callus formation (Table 3).

### 2.3. Embryogenesis in the Microspore Culture of a Turnip Variety with Microsporogenesis Disorders

**Figure 2:**
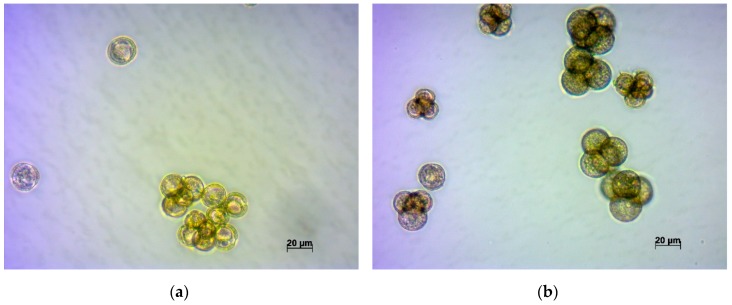

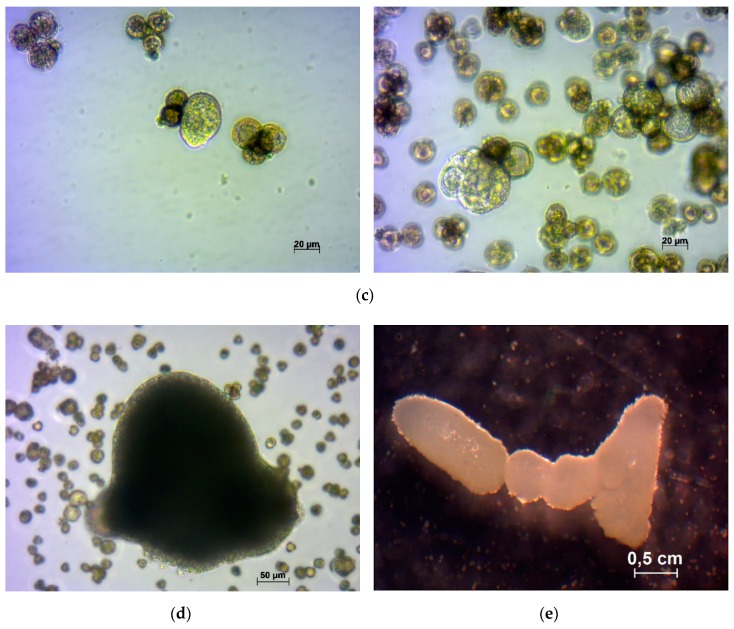
Development of microspores of turnip variety ‘Ronde witte roodkop herfst’ in the in vitro culture: (**a**) Microspores at the beginning of incubation; (**b**) microspores at day 3 of incubation; (**c**) development of microspores at day 7 of incubation; (**d**) turnip embryo developed from microspores, day 14 of incubation; and (**e**) fused turnip embryos developed from microspores (possibly non-separated ones), day 21 of incubation.

A separate study was carried out on the variety ‘Ronde witte roodkop herfst’, a distinctive feature of which was non-separation of microspores after division (Figure 2). Microscopic observation of in vitro microspores, which failed to separate from tetrads, was carried out, focused on their development. It was noted that they developed differently. Sister microspores were found to develop into:

(a) small trilobed cells, which finally died;
(b) cells which followed the original gametophytic route by dividing asymmetrically and producing generative and vegetative nuclei typical of pollen grains, non-embryogenic structures;
(c) microspores that underwent a few divisions, with enlarged cells of variable sizes that finally became arrested; and
(d) embryogenic structures that grew to globular, heart, torpedo, and cotyledonary stages.

Such observations confirm the strong influence of plant genotype on in vitro embryogenesis. The stage of microspore development and all conditions of cultivation were identical, only the genotype of sister microspores was different, as each microspore received a unique haploid set from the mother cell chromosomes, and the combination of chromosomes finally determined the manner of each microspore’s development. The embryos that formed from non-separated microspores were often fused.

### 2.4. A Study on Microspore Development in the In Vitro Culture

**Figure 3:**
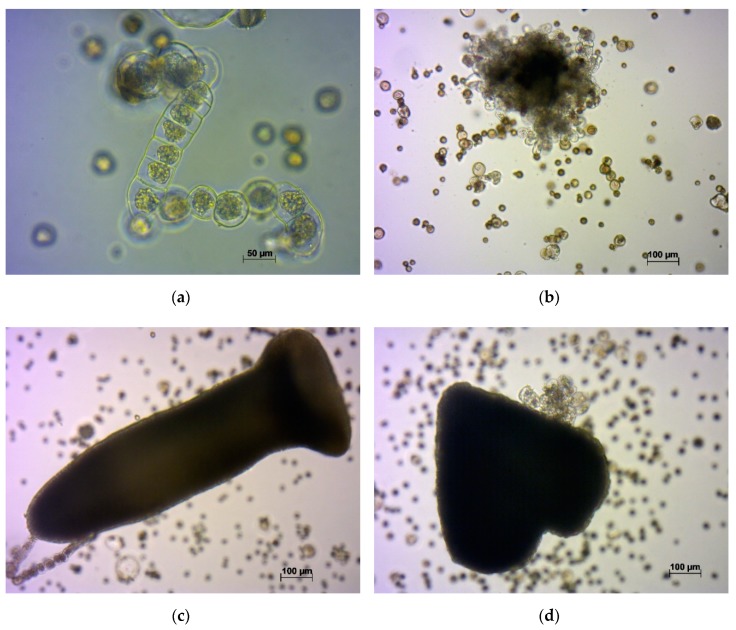
The in vitro development of isolated microspores: (**a**) Suspensor-like structure; (**b**) callus; (**c**) embryo formed from a suspensor-like structure; and (**d**) embryo developed from a microspore without formation of a suspensor-like structure.

The development of turnip microspores into embryos occurred either by direct embryo formation, or by a suspensor-like structure formation (Figure 3). The embryos that formed from suspensor-like structures lagged in development. This situation has previously been noted for rapeseed [18], broccoli [19], and Chinese cabbage [16].

### 2.5. Production of Doubled Haploid (DH) Plantlets

**Figure 4:**
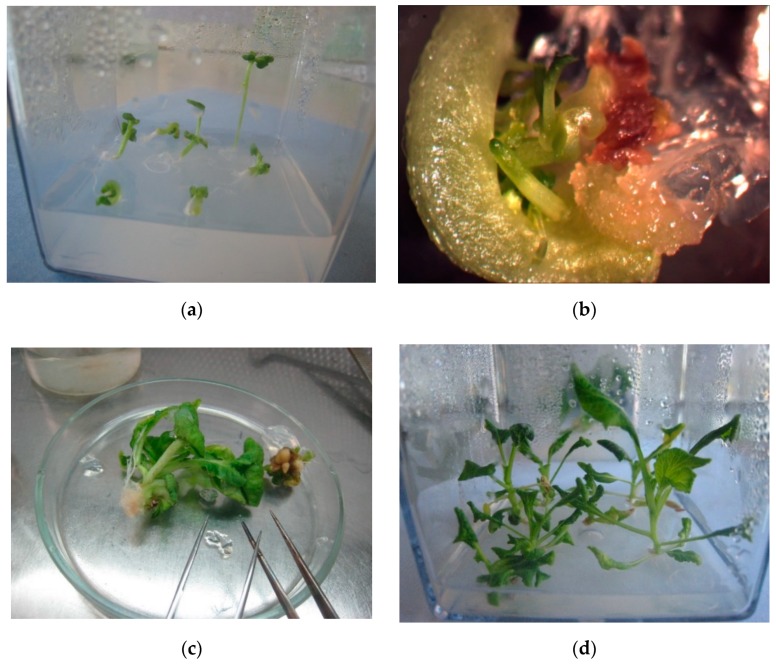
Embryo development into regenerated plantlets: (**a**) Plant regeneration directly from microspore-derived embryos on culture medium without plant growth regulators; (**b**) secondary embryos development from microspore-derived embryos on culture medium supplemented with 1 mg/L benzylaminopurine (BAP) and 0.05 mg/L gibberellic acid (GA); (**c**) cutting off the plantlets from the explant (microspore-derived embryo); and (**d**) plantlets rooting.

Microspore culture is an important method for the production of microspore-derived plants, haploids or doubled haploids. It is very important to obtain as many doubled haploid plants as possible for research and breeding purposes. Plantlets obtained as the result of germination of secondary embryos developed from primary embryos on the medium with plant growth regulators (1 mg/L benzylaminopurine (BAP) and 0.05 mg/L gibberellic acid (GA)) were spontaneous diploids in 71.9% of cases, whereas the plantlets obtained directly from primary embryos were haploid in more than 60% of cases (see Figure 4 and Table 4).

### 2.6. Ploidy Level Determination

**Figure 5:**
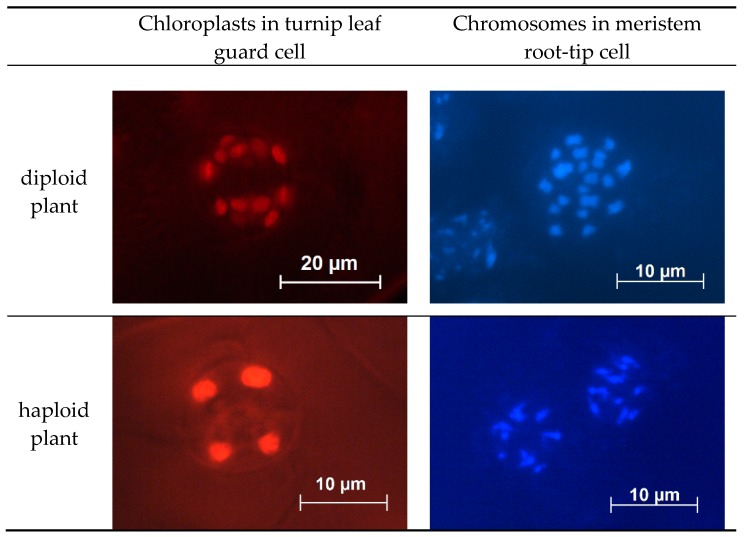
Chloroplast and chromosome numbers in cells of haploid and diploid turnip plants.

The simplest indirect method for determining ploidy level by counting the number of chloroplasts in epidermal tissue guard cells was used. Each guard cell of diploid and doubled haploid plants had 5–6 chloroplasts, unlike haploid plant cells, which contained 2–3 chloroplasts. Chromosome counting in meristem root-tip cells, stained with Dapi (Figure 5) was performed to confirm the relationship between the number of chromosomes and the number of chloroplasts in the turnip leaf guard cells. Diploid and doubled haploid plants had 20 chromosomes, while haploid plants had 10 chromosomes.

Ploidy levels of plantlets were determined, and a high frequency of diploids was shown in all experimental turnip cultivars obtained on the MS medium supplemented with BAP 1 mg/L and GA 0.05 mg/L (Table 4)

## 3. Discussion

Responsiveness in the microspore culture depends on the genotype. Therefore, a small yield of embryos means the loss of some genotypes, which could be useful for breeding. To solve this problem, data from the literature were used alongside the results of our own research to develop a protocol for working with different cabbage crops. The culture of turnip microspores was characterized by an unstable yield of embryos from the same variety, comparing experiments carried out in the same way but on different days. The reason for this could be due to the age of donor plants or the ratio of microspores from each plant in the total sample, as not only the plant variety affects the efficiency of embryogenesis, but also the genotype of an individual plant; this effect has been well-described in an article by Chuong et al. [20]. Despite the difference in the absolute number of embryos of each variety in different experiments, correlations of reactions of genotypes to factors were always preserved. It was shown in the present work that the use of different pH makes it possible to obtain the greatest number of embryos from each sample, where the increase in yield can be up to 66% (for the Roots variety). Despite the fact that classical microspore culture protocols use a medium with pH 5.8, the first factor to increase the embryo yield for some recalcitrant cultures is to use a medium with a higher pH [4]. The use of activated charcoal always positively influenced embryo development, increasing the yield in some varieties more than two-fold. During the cultivation process, many microspores die. Consequently, the substances released by them adversely affect the development of embryos; however, the use of activated charcoal reduces the effect of these toxic substances [12,21]. In some turnip genotypes studied both embryos and calluses developed from microspores were observed; this phenomenon is not common for plants of the Brassica family when exogenous auxins are not added to the culture medium, although such an effect has been is described in radish [22]. As a rule, such callus-like structures do not develop into embryos in the microspores culture; therefore, it is important to prevent the transition of microspores to callus and to induce their direct development into embryos. The well-known positive effect of flower bud cold pre-treatment on the development of embryos in microspore culture [5,23,24] was confirmed in experiments, with an increase in the number of embryos in the microspore culture and a simultaneous decrease in callus formation.

Many researchers have noted that obtaining embryos does not mean that they will all develop in normally plants [17,25], and may form doubled haploid plants. In experiments conducted with turnip, we showed that most of the formed embryos could be successfully developed into plants on solid medium with BAP 1 mg/L and GA 0.05 mg/L. Cultivation of embryos on this medium led to the formation of secondary embryos, which as a rule developed into normal plants without deformation.

To obtain the maximum number of doubled haploids, which can be used directly for breeding purposes, researchers use various approaches; most often, colchicine is applied to microspores at the early stages of cultivation [26,27].

Among plantlets, which were developed from secondary embryos, more than 70% plants were detected as spontaneous doubled haploids. The reason for this seems to be through endomitosis or endoreduplication [28]. Spontaneous doubled haploid development has been described by other researchers, but with frequencies ranging from 10% to 30% [29,30,31].

Apparently, the time required for the formation of secondary embryos allows chromosomes in cells to double and, possibly, cells with a doubled composition of chromosomes to develop into embryos more quickly.

Among the studied varieties of turnip, one was unique (‘Ronde witte roodkop herfst’) a distinctive feature of which was the non-separation of microspores after division, which remained stuck together as in Typha and Calluna. This developmental disability, however, did not prevent the successful pollination of these plants and the development of microspores in the culture of microspores into embryos. Through this variety, it was possible to observe the various development of microspores formed from one mother cell. Further study of this variety and its doubled haploids may serve as very interesting for understanding the factors that influence the development of plant microspores.

This work demonstrates that the selection of individual cultivation conditions makes it possible to obtain the maximum number of embryos in the microspore culture of various turnip genotypes, even for a genotype with a genetic disorder in microspore development.

## 4. Materials and Methods

### 4.1. Plant Material and Plants Growing Conditions

Four turnip accessions from the Vavilov All-Russian Institute of Plant Genetic Resources collection have been evaluated (Table 5). 

Donor plants were grown from vernalized turnip roots, in a climatic chamber (at 19 °C, 16 h day /8 h night photoperiod; light level 9000 lux, lamp: horturion HPS, 600 W 220 V E40). Cytological observation of microspore development stages in selected flower buds was carried out. For microspore and pollen visualization, the differential staining technique [32] and an Axio Imager A2 microscope were used. Only the buds containing microspores at the late stage of pollen development (uninucleate stage) were used.

### 4.2. Microspore Culture

Flower buds 2–3 mm long (late uninucleate stage microspore development) were collected from plants at the initial stage of flowering. For low temperature pretreatment, inflorescences were collected and put into a plastic Petri dish containing filter paper with 5 mL of sterile water, and then were kept in darkness at 6 °C for 2 days. Flower buds were disinfested with 70% (*v*/*v*) ethanol for 5 min, 15% (*w*/*v*) sodium hypochlorite for 10 min, and rinsed with sterile distilled water (SDW) for 10 min. Sterile buds were crushed in ½ NLN-13 modified medium (without potato extract) [1] with 13% sucrose (*w*/*v*) without potato extract and plant growth regulators, at pH 5.8. The suspension was filtered through a 60 μm mesh. Then, the microspore suspension was centrifuged at 100 *g* for 5 min, the supernatant was removed, and ½ NLN-13 was added to each tube. This step was repeated twice, and then filter-sterilized and modified NLN-13 liquid medium (pH from 5.8–6.6, depending on the experiment) was added to the microspores to achieve the final suspension density of microspores from 1 bud per 1 mL of medium. The microspore suspension was then poured into 60-mm Petri dishes (5 mL per Petri dish), and the cultures were kept in darkness at 32 °C for 48 h. After that, incubation continued at 25 °C on a gyratory shaker at 60 rpm until the initiation of embryos. A minimum of 3 plants of each variety were used in each experiment. Each experiment was performed 2–3 times in four variant replicates (4 Petri dish for each variant).

### 4.3. The Influence of Plant Genotype on the Induction of Embryogenesis in Microspore Culture

Buds were collected from plants of each variety at the same phase of plant development (initial stage of flowering). Microspores were cultured in ½ NLN-13 medium supplemented with 13% (*w*/*v*) sucrose at pH 5.8.

### 4.4. The Influence of the Medium pH and Activated Charcoal Addition on the Induction of Embryogenesis in Microspore Culture

Microspores were cultured in ½ NLN-13 medium supplemented with 13% (*w*/*v*) sucrose at pH 5.8, 6.2 or 6.6. In experiments with AC, 250 μL of the autoclaved 1% suspension of activated charcoal in 0.5% (*w*/*v*) agarose was added to a Petri dish before adding the main liquid medium.

### 4.5. Plant Regeneration

Embryos at different formation stages (viz. globular, heart-shaped and torpedo-shaped) were transferred onto Petri dishes containing a solid medium. For direct plant regeneration from microspore derived embryos, the MS [33] medium supplemented with 2% (*w*/*v*) sucrose and 3 g/L Phytagel (Sigma-Aldrich, Co, St. Louis, MO, USA) was used. To obtain the secondary embryos, the microspore-derived embryos were transferred into the MS medium supplemented with 2% (*w*/*v*) sucrose, 3 g/L Phytagel, 1 mg/L benzylaminopurine (BAP) and 0.05 mg/L gibberellic acid (GA). The regenerated plantlets and secondary embryos were transferred to the ½ MS medium supplemented with 2% (*w*/*v*) sucrose and 3 g/L Phytagel for root development. Fluorescent illumination at 25,000 lux, 14 h photoperiod, and 25 °C temperature were provided in the growing chamber for cultivation.

### 4.6. Plantlets Growing

Plantlets with normally-developed leaves and root system were transferred to vegetation vessels filled with a mixture of peat and perlite (7:3). They were covered by perforated plastic cups for the best adaptation to the in vivo conditions. Light and temperature conditions were the same as described in Section 4.1.

### 4.7. Ploidy Level Determination

The ploidy level in regenerated plantlets was determined by counting chloroplasts in guard cells. The lower epidermis was peeled, rinsed in distilled water, placed onto a microscope slide in a water drop, and covered with a cover slip. Observations were made using a fluorescent Axio Imager A2 microscope with BP 490 and 515 filter sets. At least 10 pairs of stomata guard cells of each plant were photographed and chloroplasts counted (chloroplasts exhibiting very strong autofluorescence in red, with a peak of approximately 680 nm). Plant ploidy level was also determined by chromosome counting in the apical root meristems stained with Dapi (Axio Imager A2 microscope with fluorescence) [34].

### 4.8. Statistical Analysis

Statistical analysis was performed using one-way ANOVA (analysis of variance), factorial ANOVA, and means were compared using Duncan’s multiple range test (DMRT) with a probability of 95% The statistical analyses were carried out using Statistica 8.0 (Statsoft www.statsoft.com).

## Figures and Tables

**Table 1:** Embryo and callus development in the microspore culture isolated from flower buds of different turnip varieties.

Variety	Mean Embryo Numbers Per Petri Dish, pcs	Mean Callus Number Per Petri Dish, pcs
Ronde witte roodkop herfst	5.0 a *	1.3 a
Snow ball	3.0 a	3.0 b
York Globe	3.3 a	2.5 b
Roots	20.7 b	0 c

* Variants marked with the same letter do not have a significant difference with a probability of 95% for Table 1, Table 2 and Table 3.

**Table 2:** Influence of acidity of the medium and AC presence on the development of embryo from microspores of the Roots variety.

pH Level	AC Presence/Absence in the Medium	Mean Embryo Numbers Per Petri Dish, pcs.
5.8	+	17.2 a
	−	15.6 a
6.2	+	23.0 b
	−	16.3 a
6.6	+	53.4 c
	−	25.6 ab

**Table 3:** Influence of flower bud cold pre-treatment on embryo development in turnip microspore culture.

Variety	pH Level *	Flower Buds Pre-Treated at 10 °C for 2 Days	Mean Embryo Numbers Per Petri Dish, pcs	Mean Callus Numbers Per Petri Dish, pcs
Ronde witte roodkop herfst	6.2	+	8.7 a	0 a
		−	2.7 b	4.1 b
Snow ball	6.2	+	7.9 ad	5.6 b
		−	0 c	31.2 c
York Globe	6.6	+	5.7 d	1.5 d
		−	1.2 e	20.1 e
Roots	6.6	+	65.7 f	0 a
		−	50.8 g	0 a

* pH level was optimal for each variety.

**Table 4:** Ploidy level of plantlets obtained from primary microspore-derived embryos and secondary turnip embryos.

	Number of Observed Plantlets, psc	Diploid Plant, %	Haploid Plant,%
Plantlets obtained directly from primary embryos	215	40.0	60.0
Plantlets obtained as the result of germination of secondary embryos	203	71.9	28.1

**Table 5:** Donor cultivars of turnip.

VIR Catalog Number	Name	Country
k-995	Ronde witte roodkop herfst	Netherlands
k-1255	Snow ball	India
k-1281	York Globe	Australia
k-1380	Roots	USA

## Supplementary Materials

The following are available online at https://www.mdpi.com/2223-7747/9/2/278/s1, Table S1: Influence of acidity of the medium and AC presence on the development of embryo from microspores of the Ronde witte roodkop herfst variety, Table S2: Influence of acidity of the medium and AC presence on the development of embryo from microspores of the Snow ball variety, Table S3: Influence of acidity of the medium and AC presence on the development of embryo from microspores of the York Globe variety.
