# Supplementary material for: Effects of Genotype and Culture Conditions on Microspore Embryogenesis and Plant Regeneration in Brassica Rapa ssp. Rapa L
Source: Plants (Basel). 2020 Feb 21;9(2):278. doi: 10.3390/plants9020278 (PMC7076648; doi:10.3390/plants9020278)
Supplement: Supplementary file 1 [file plants-09-00278-s001.pdf]

Table S1. Influence of acidity of the medium and AC presence on the development of embryo from microspores of the Ronde witte roodkop herfst variety.

pH level	AC presence/absence in the medium	Mean embryo numbers per Petri dish, pcs	Mean callus numbers per Petri dish, pcs
5.8	+	3.5a*	3.2a
	-	3.1 ac	4.3 a
6.2	+	4.7b	3.8a
	-	2.2c	4.2a
6.6	+	0.7d	3.0a
	-	0e	0b

*Variants marked with the same letter do not have a significant difference with a probability of 95% for Tables S1–3.

Table S2. Influence of acidity of the medium and AC presence on the development of embryo from microspores of the Snow ball variety.

pH level	AC presence/absence in the medium	Mean embryo numbers per Petri dish, pcs	Mean callus numbers per Petri dish, pcs
5.8	+	3.2a	4.8a
	-	2.7a	4.5a
6.2	+	3.7a	27.2b
	-	0b	10.2c
6.6	+	1.2c	10.3c
	-	0b	5.2a

Table S3. Influence of acidity of the medium and AC presence on the development of embryo from microspores of the York Globe variety.

pH level	AC presence/absence in the medium	Mean embryo numbers per Petri dish, pcs	Mean callus numbers per Petri dish, pcs
5.8	+	1.7a	2.1a
	-	1.5 a	2.2 a
6.2	+	1.9a	5.9b
	-	1.8a	10.2b
6.6	+	4.2b	25.1c
	-	2.8a	20.8d

For determining the optimal pH for each variety, the number of formed embryos, as well as the amount of callus, were taken into consideration, since callus is the result of the microspores development, it means there is a chance to change the process of callus formation into embryo formation.
